# Supplementary figures and images for: Development of AI-Augmented optimization technique for analysis & prediction of modal mix in road transportation
Source: PLoS One. 2023 Nov 2;18(11):e0288493. doi: 10.1371/journal.pone.0288493 (PMC10621837; doi:10.1371/journal.pone.0288493)

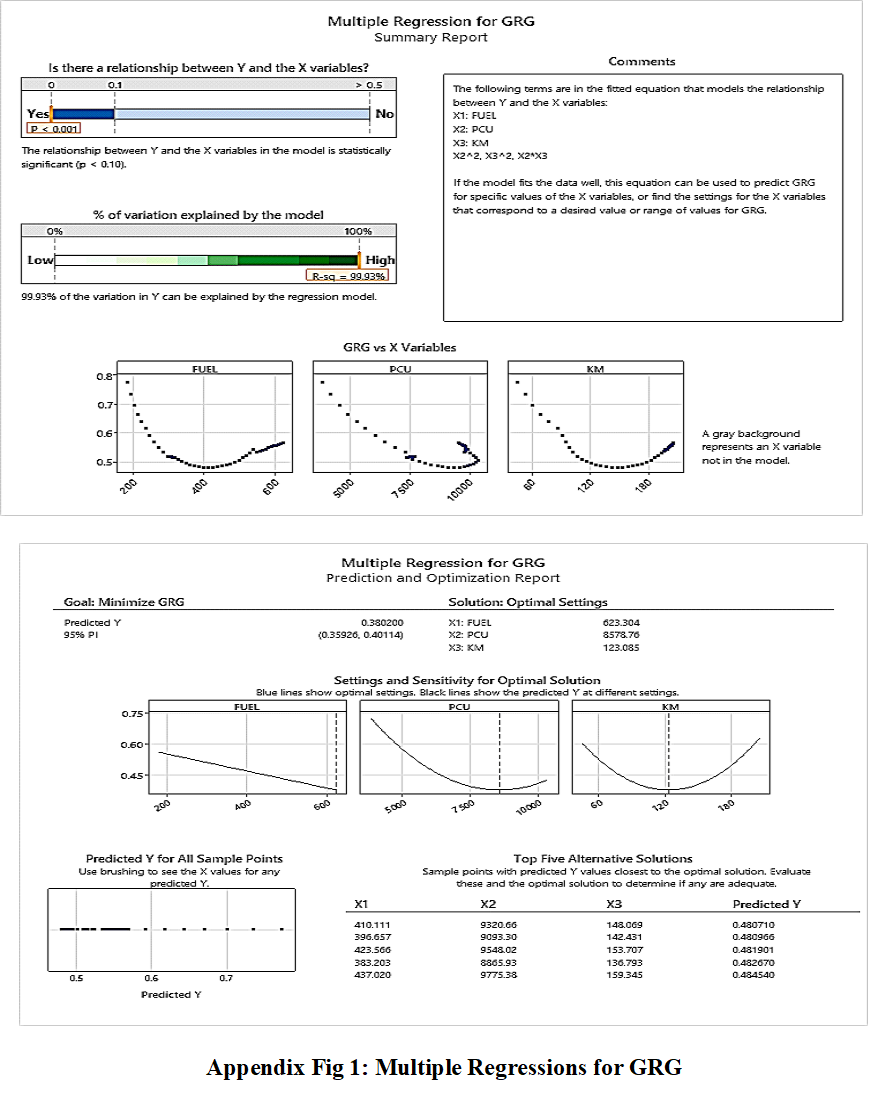

Supplement: S1 Fig — (TIF) [file pone.0288493.s001.tif]

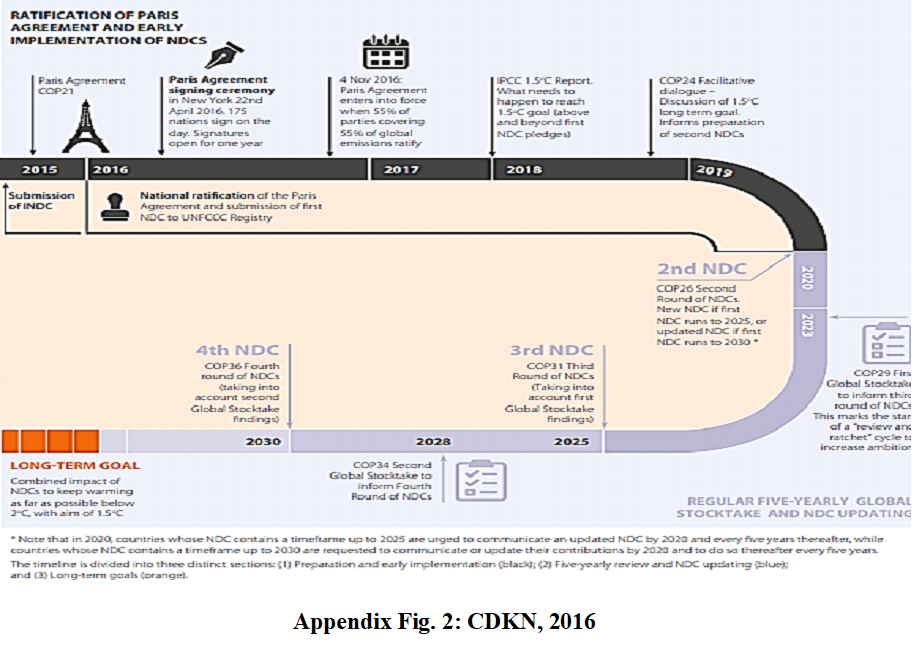

Supplement: S2 Fig — (TIF) [file pone.0288493.s002.tif]

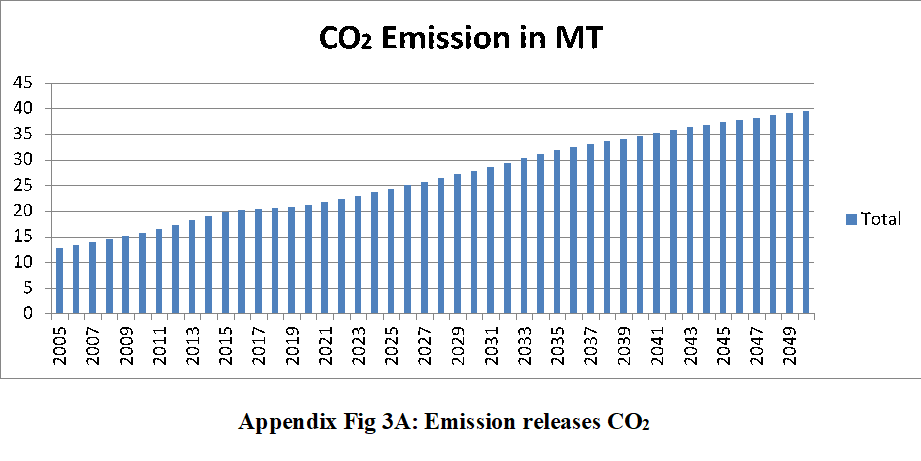

Supplement: S3 Fig — (TIF) [file pone.0288493.s003.tif]

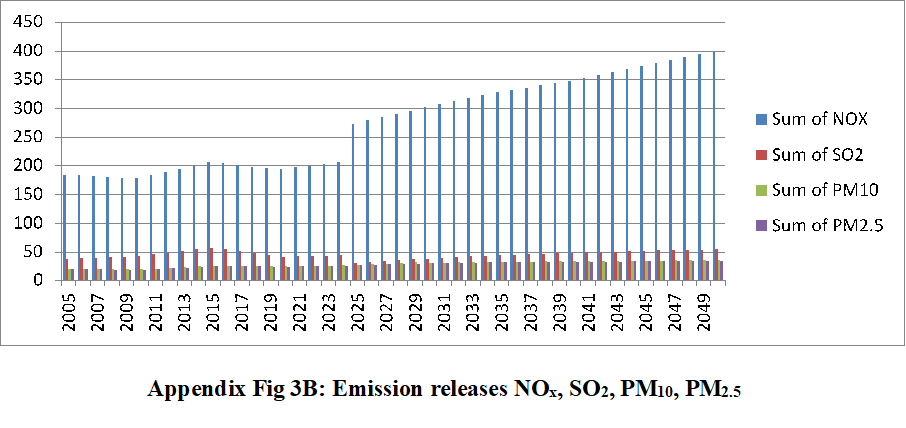

Supplement: S4 Fig — (TIF) [file pone.0288493.s004.tif]

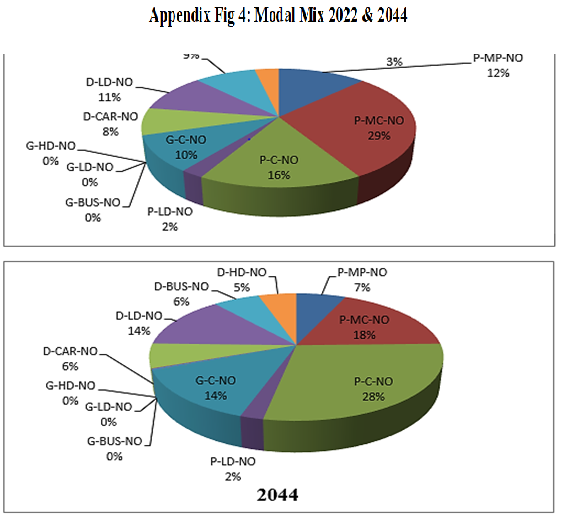

Supplement: S5 Fig — (TIF) [file pone.0288493.s005.tif]

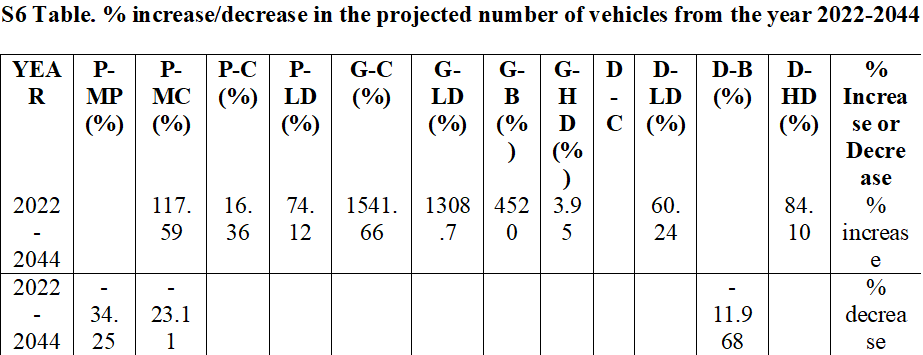

Supplement: S1 Table — (TIF) [file pone.0288493.s006.tif]

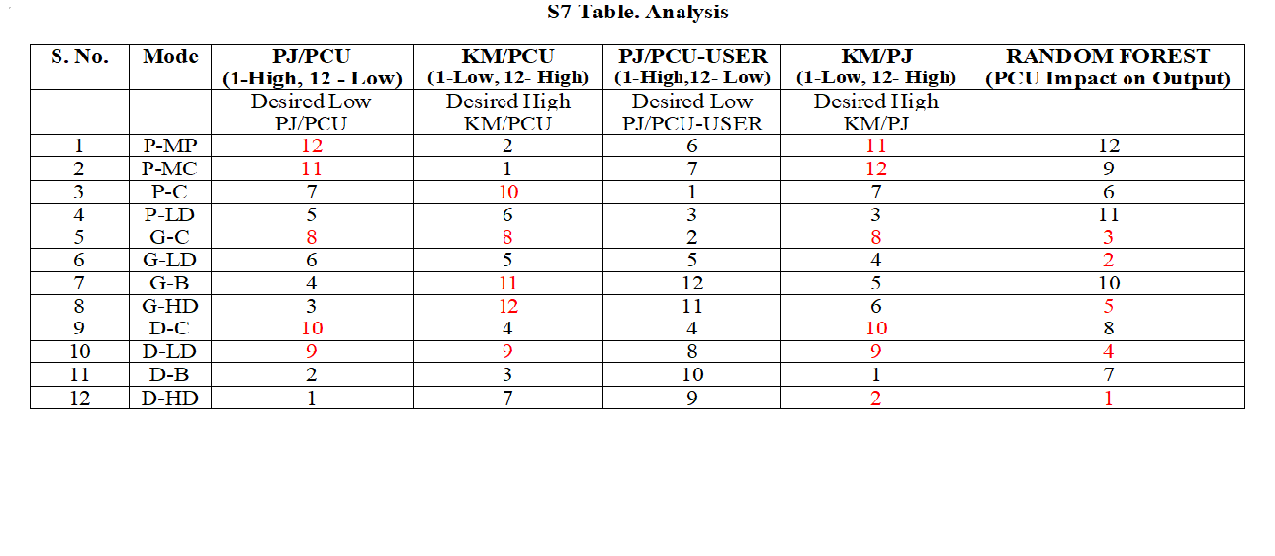

Supplement: S2 Table — (TIF) [file pone.0288493.s007.tif]

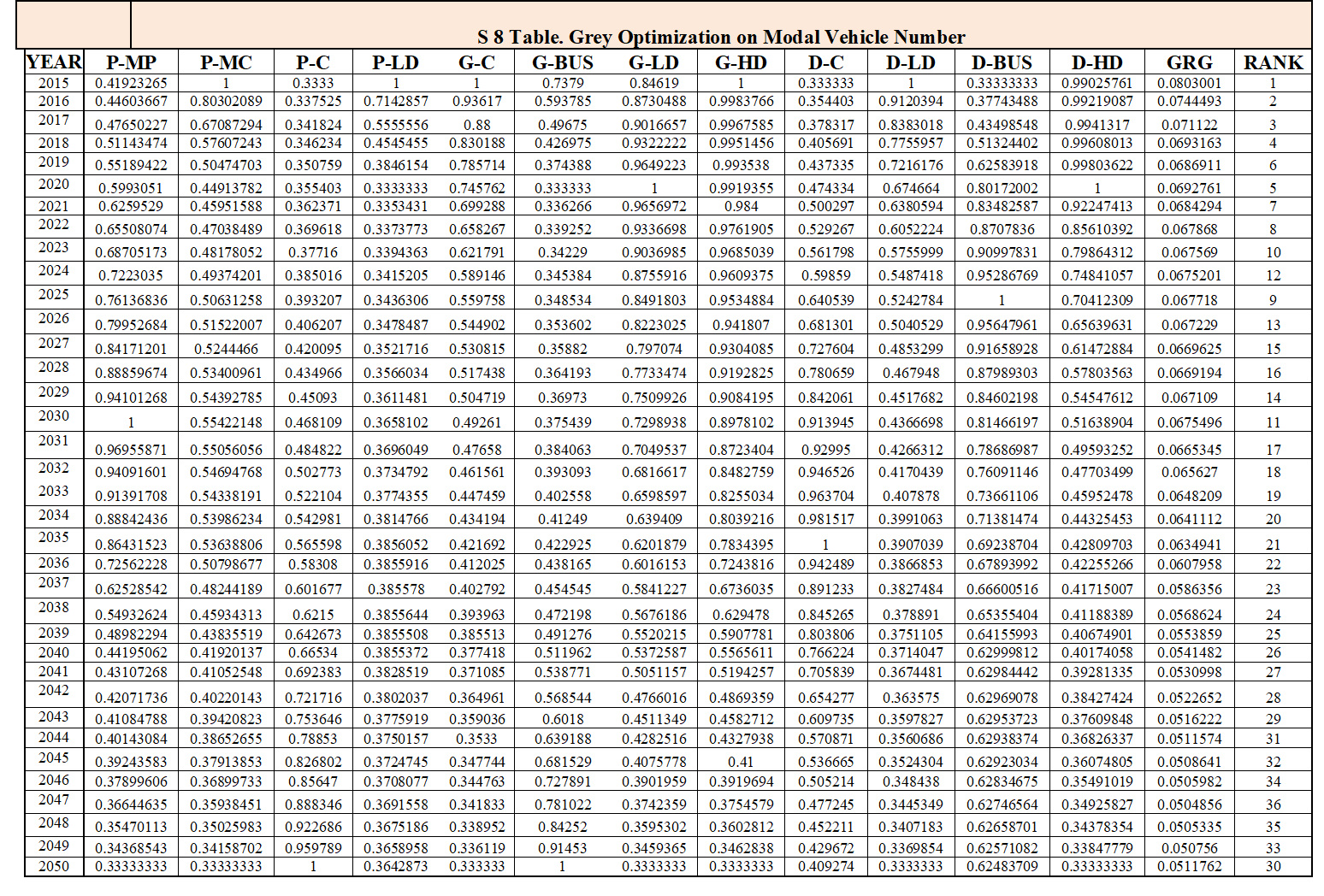

Supplement: S3 Table — (TIF) [file pone.0288493.s008.tif]
