# Supplementary material for: Development of AI-Augmented optimization technique for analysis & prediction of modal mix in road transportation
Source: PLoS One. 2023 Nov 2;18(11):e0288493. doi: 10.1371/journal.pone.0288493 (PMC10621837; doi:10.1371/journal.pone.0288493)
Supplement: Attachment — Submitted filename: Response to reviewer.doc [file pone.0288493.s009.doc]

June 1, 2023

**Reference:**	PONE_D-23-04895, Development of AI-Augmented Optimization Technique for Analysis & Prediction of Modal Mix in Road Transportation for PLOS ONE

Dear Dr. Muhammad Kamran Khan,

Kindly refer to the email dated April 18, 2023, regarding the revision requirement of the referenced article, I am pleased to submit the revised article with this letter, and compliance is mentioned below:

**Journal Requirements:**

| S.No. | Requirement | Compliance |
| --- | --- | --- |
| 1. | Please ensure that your manuscript meets PLOS ONE's style requirements, including those for file naming. The PLOS ONE style templates can be found at  <https://journals.plos.org/plosone/s/file?id=wjVg/PLOSOne_formatting_sample_main_body.pdf> and  <https://journals.plos.org/plosone/s/file?id=ba62/PLOSOne_formatting_sample_title_authors_affiliations.pdf> | Templates for both title & author affiliation along with the main body followed in the manuscript submitted. |
| 2. | We note that you have stated that you will provide repository information for your data at acceptance. Should your manuscript be accepted for publication, we will hold it until you provide the relevant accession numbers or DOIs necessary to access your data. If you wish to make changes to your Data Availability statement, please describe these changes in your cover letter and we will update your Data Availability statement to reflect the information you provide. | Data is already been made available in the paper and changes in the Data Availability statement are made accordingly. |

**Reviewer #1 Comments:**

| S.No. | Requirement | Compliance |
| --- | --- | --- |
| 1. | In the abstract, please include 2-3 special quantitative achievements from the findings of this study in the context of the environment by combining the research objectives and problems. Please limit your abstract to 250 words. Check spellings for many words that are misspelled or written in haste. | Revised and Complied |
| 2. | The introduction section needs a few more sentences to strengthen the article, and please include the research problem, objective, and novelty in the last paragraph of the Introduction section. | Revised and Complied |
| 3 | Include a few more sentences at the beginning of the introduction explaining your paper's contribution to the environment, climate change impact, and sustainability, as well as your attempts to deal with or present solutions to a specific problem/s and your unique contribution to this research paper. | Revised and Complied |
| 4 | Please also present the methodology section in a concise graphical format. | Revised and Complied |
| 5 | The literature review section is very weak; please revise it. | Revised and Complied |
| 6 | Please present your literature review in the form of a SmartArt chart. | Revised and Complied |
| 7 | Just after the Methodology, please mention the societal benefits of your research in terms of evaluating its key determinant. | Revised and Complied |
| 8 | In 500-750 words, explain research problems, solutions, and the theoretical contribution of your study in the "Results" section. I found that the literature section is a little weak, shift your study a little more towards environment friendly and sustainability, therefore it requires more studies to be reviewed therefore I suggest you to include the following work: <https://doi.org/10.1016/j.strueco.2022.04.003> <https://doi.org/10.1016/j.envres.2022.112848> <https://doi.org/10.1108/MD-03-2022-0407> <https://doi.org/10.1007/s11356-022-20567-6> <https://doi.org/10.1177/09763996211041215> <https://doi.org/10.1007/s11356-023-25574-9> <https://doi.org/10.1007/s11356-021-15421-0> | Revised and Complied. All mentioned papers are included in the paper. |

I hope to have replied to all revision requirements and sincerely look forward to the acceptance of this paper for publication in your esteemed Journal.

Sincerely,

Huma Rauf

Department of Business & Engineering Management,

Sir Syed CASE Institute of Technology (SSCIT), B-17

Islamabad 44400, Pakistan
